# Examining links between anxiety, reinvestment and walking when talking by older adults during adaptive gait

**DOI:** 10.1007/s00221-015-4445-z

**Published:** 2015-09-24

**Authors:** William R. Young, Mayowa Olonilua, Rich S. W. Masters, Stefanos Dimitriadis, A. Mark Williams

**Affiliations:** Department of Clinical Sciences, Brunel University, London, UK; Department of Life Sciences, Brunel University, London, UK; Institute of Human Performance, University of Hong Kong, Hong Kong, China; Te Oranga School of Human Development and Movement Studies, University of Waikato, Hamilton, New Zealand

**Keywords:** Stops walking when talking, Movement self-consciousness, Conscious motor processing, Attention, Fear of falling, Falls, Working memory

## Abstract

Falls by older adults often result in reduced quality of life and debilitating fear of further falls. Stopping walking when talking (SWWT) is a significant predictor of future falls by older adults and is thought to reflect age-related increases in attentional demands of walking. We examine whether SWWT is associated with use of explicit movement cues during locomotion, and evaluate if conscious control (i.e. movement specific reinvestment) is causally linked to fall-related anxiety during a complex walking task. We observed whether twenty-four older adults stopped walking when talking when asked a question during an adaptive gait task. After certain trials, participants completed a visuospatial recall task regarding walkway features, or answered questions about their movements during the walk. In a subsequent experimental condition, participants completed the walking task under conditions of raised postural threat. Compared to a control group, participants who SWWT reported higher scores for aspects of reinvestment relating to conscious motor processing but not movement self-consciousness. The higher scores for conscious motor processing were preserved when scores representing cognitive function were included as a covariate. There were no group differences in measures of general cognitive function, visuospatial working memory or balance confidence. However, the SWWT group reported higher scores on a test of external awareness when walking, indicating allocation of attention away from task-relevant environmental features. Under conditions of increased threat, participants self-reported significantly greater state anxiety and reinvestment and displayed more accurate responses about their movements during the task. SWWT is not associated solely with age-related cognitive decline or generic increases in age-related attentional demands of walking. SWWT may be caused by competition for phonological resources of working memory associated with consciously processing motor actions and appears to be causally linked with fall-related anxiety and increased vigilance.

## Introduction

Falls are a leading cause of injury in older adults, reducing quality of life and increasing the risk of further falls (Rubenstein 2006). The process of observing whether an older adult stops walking when talking (SWWT; Lundin-Olsson et al. 1997) is an efficient, cost-free clinical tool that can predict falls (Beauchet et al. 2009). Gait decrements observed when older adults concurrently walk and talk are considered to be a measure of dual-task costs incurred when the respective tasks compete for attentional resources (Montero-Odasso et al. 2012; Verghese et al. 2007; Ayers et al. 2014). There is general consensus in the literature that SWWT highlights either age-related cognitive decline and/or a generic increase in the attentional cost of regulating posture (Boisgontier et al. 2013). We propose that SWWT is underpinned by a propensity to focus attention internally in order to consciously process movements that are normally automatic (i.e. walking); a phenomenon termed ‘movement specific reinvestment’ (Masters 1992; Masters and Maxwell 2008).

According to the theory of reinvestment, people in the early stages of learning ontogenetic skills are heavily reliant on declarative information about how to execute specific movements. In an attempt to consciously control movements, they unwittingly freeze redundant degrees of freedom within the kinematic chain (Bernstein 1967; Masters and Maxwell 2008). As a result of practice, declarative information is consolidated into procedural knowledge and movement execution becomes more automatic, placing fewer demands on cognitive resources. This progression corresponds with gradual freeing of the degrees of freedom, such that expert performers can exploit the flexibility of the human motor system (Bernstein 1967; Masters and Maxwell 2008). Reinvestment is a term used to describe situations in which individuals who are able to execute a motor task with relative autonomy reinvest cognitive effort in consciously controlling specific aspects of performance that are otherwise carried out subconsciously (often when experiencing high levels of performance anxiety) (Masters and Maxwell 2008).

The concept of movement specific reinvestment has already been broached in the literature concerning the control of posture and gait in older adults. Wong et al. (2008, 2009) asked older adults to perform walking tasks of varying difficulty (carrying no cup, an empty cup, or a cup filled with water). When categorising participants as being either ‘fallers’ or ‘non-fallers’, the authors showed that fallers self-reported higher movement specific reinvestment than non-fallers, and provided more accurate responses to questions about the position of their body during the walking trial, demonstrating greater awareness of internal factors. However, no significant differences were found between fallers and non-fallers in measures reflecting awareness of external environmental factors (Wong et al. 2009).

It is difficult to disassociate the broad concept of movement specific reinvestment from widely reported generic age-related increases in the attentional demands of walking. The two concepts are not mutually exclusive. The propensity for movement specific reinvestment is commonly measured using the Movement Specific Reinvestment Scale (MSRS) (Masters et al. 2005; Masters and Maxwell 2008), which evaluates two distinct dimensions of reinvestment, *movement self*-*consciousness* (MSRSms-c) and *conscious motor processing* (MSRScmp). MSRSms-c reflects a propensity to monitor the way a movement is executed, whereas MSRScmp reflects a tendency to consciously control movement mechanics. Although the MSRS is often considered a unidimensional construct (Malhotra et al. 2012), the two dimensions may play distinct roles with respect to the allocation of attention during motor performance.

According to the theory of reinvestment (Masters 1992; Masters et al. 1993; Masters and Maxwell 2008), conscious motor processing is a function of the use of explicit information to support motor execution, which inevitably places demands on aspects of working memory responsible for processing phonetic information [i.e. the phonological loop (Baddeley 1986, 2007)]. Like conscious control of movement, verbally responding to questions when walking inevitably places demands on phonetic aspects of working memory. Although researchers have measured specific gait characteristics during walking when talking tasks, measuring SWWT has the advantage of offering a clear binary method for categorising older adults. The first objective of the current study was to determine if older adults who SWWT report a greater propensity to consciously control their walking actions (as measured by the MSRScmp) than they do movement self-consciousness (as measured by the MSRSms-c), and whether they do so more than older adults who do not SWWT.

Providing that sufficient cognitive resources are available, it is possible that some older adults can consciously control their gait and verbally respond to a question. Therefore, we made the additional prediction that higher MSRScmp scores in older adults who SWWT will not be preserved when accounting for a measure of cognitive function.

**Fig. 1:**
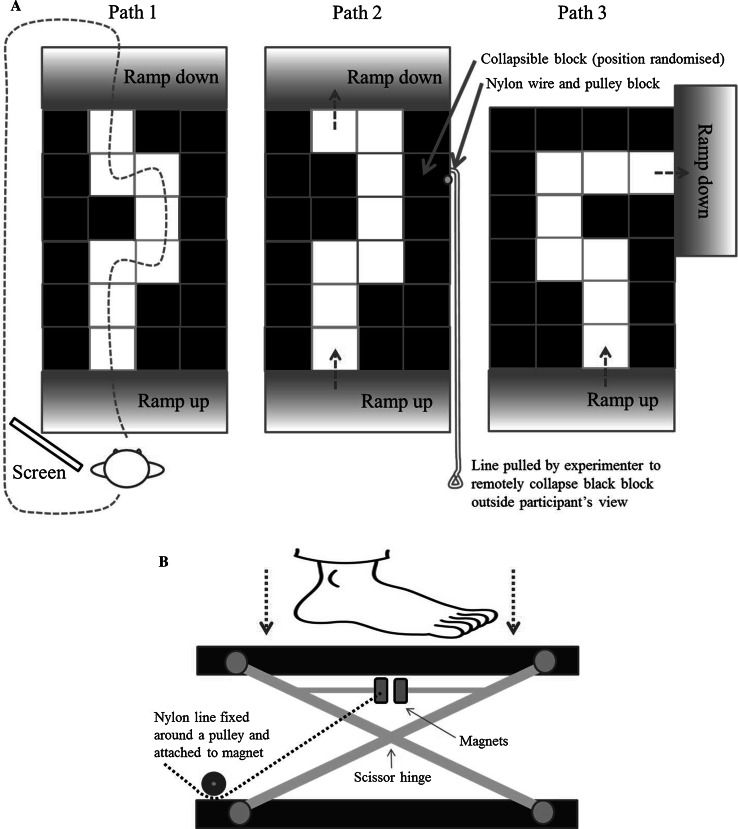
**a** Schematic of path sequences and direction of walking. The *dotted line* in Path 1 indicates the route that participants took (returning on the left side of the walkway in all trials). The small *dashed arrows* in Paths 2 and 3 indicate the first and last blocks in the walkway along with the direction of walking. The *solid grey arrows* in Path 2 show example locations of collapsible *black* *blocks*, along with the approximate layout of the nylon wire that was used by experimenters to ‘trigger’ the block to collapse during false trials. For the VSR task, participants were presented with pen-and-paper task where, directly after walking, they were asked to mark the position of the *white* *path* on a blank 4 × 6 grid with the start position of the walkway identified. The VSR task was scored using the following rules: (a) 1 point for each block correctly marked; (b) subtract 0.5 points for each block where no attempt was made to mark that area of the path; (c) 1 point for each turn in the walkway correctly identified. Finally, as there were eight white blocks in every path sequence, if a participant marked more than eight blocks, 1 point was deducted for every block marked over the total of eight. Possible scores ranged from −8 to +10. **b** A cross-sectional schematic of the collapsible block mechanism. The distance between the magnets was adjusted so that experimenters only needed to impart a small amount of force to cause the support surface to fall (vertical distance of fall = 18 cm)

In a similar fashion to Wong et al. (2009), we also sought to determine participants’ awareness of internal and external factors. Specific criticisms can be made of the questions used by Wong et al. (2009) regarding participants’ external awareness. For example, a correct response to the question ‘was your body in front of, or behind, the marker when you heard the tone?’ would require a participant to be monitoring both the external information relevant to the visual marker and the auditory tone and the position of their own body. Consequently, the task probably did not solely reflect external monitoring. In the current study, we therefore assessed internal monitoring using Wong et al.’s (2009) question task, and external monitoring using a novel visuospatial recall (VSR) task (see Fig. 1). Failure to accurately recall details of the route walked was taken as a marker of propensity to allocate attention away from external task-relevant aspects of the environment. We predicted that older adults who SWWT (reporting higher MSRScmp) would perform worse on the VSR.

In older adults, movement specific reinvestment is often triggered by awareness of movement difficulties and associated fall-related anxiety (Wong et al. 2008, 2009). The motor learning and performance literature suggests that increased anxiety often leads to conscious monitoring and control of movements (for review, see Masters and Maxwell 2008), which can disrupt performance (Beilock and Carr 2001; Gray 2004; Lam et al. 2009; Masters 1992) by compromising movement fluency (Masters and Maxwell 2008; Lohse et al. 2011; Yoshie et al. 2009; Stins et al. 2011). This pathway appears to extend to motor tasks typically regulated by automatic processes, such as controlling posture (Wulf et al. 2001), so the second objective of the current study was to use our adaptive walking task to examine if increases in state anxiety were associated with increased self-reported movement self-consciousness, conscious motor processing, and scores relating to internal and external awareness.

Traditionally, common methods for inducing anxiety have included crowd pressure, opportunity for monetary gain/loss, negative performance feedback and ego stressors (e.g. Masters 1992; Liao and Masters 2002). These manipulations may serve to increase various aspects of cognitive and/or somatic anxiety, but they do not lead specifically to increased fear of falling and may, therefore, not induce representative changes in associated behaviours. Researchers studying postural control have devised an eloquent method for inducing anxiety specifically related to fear of falling, by raising the height (or perceived height) of a support platform (Carpenter et al. 1999; Adkin et al. 2000; Gage et al. 2003; Nibbeling et al. 2012; Huffman et al. 2009; Zaback et al. 2015). Anxiety-related adaptations in postural control can be broadly conceptualised as a global stiffening response, characterised by co-contractions in affecting muscle groups (for review, see Staab et al. 2013). Increased perception of postural threat has been shown to induce a so-called posture-first attention strategy during straight walking (Gage et al. 2003) (i.e. prioritising postural control over the performance of a concurrent cognitive or motor task). Furthermore, when standing on a raised platform (Threat condition), young adults self-report higher MSRS scores compared to standing at ground level (Huffman et al. 2009; Zaback et al. 2015). These studies clearly establish a causal link between anxiety and reinvestment, but only report observations in young adults during a stationary posture task (Huffman et al. 2009; Zaback et al. 2015). It is unclear whether the findings translate to the context of older adults performing an adaptive gait task that requires feedforward movement planning (Patla and Vickers 1997) and, possibly, online correction of movements. In their cross-sectional studies, Wong et al. (2008, 2009) implied a causal association between anxiety and reinvestment in older adults during a walking task. However, neither state- nor trait anxiety was measured. We need to evaluate whether increased anxiety is causally associated with increased reinvestment in older adults during adaptive gait, and to determine if either dimension of the MSRS, along with internal and external awareness, is significantly influenced. This is a significant challenge, largely because accurately comparing awareness of external factors requires visual information available within the task to be identical across experimental conditions. In this study, we describe a novel method for manipulating anxiety that meets this latter criterion and serves to induce anxiety in a manner that is specific to fear of falling.

We predict that, when faced with perceived postural threat during walking, older adults will self-report higher state anxiety, and higher conscious motor processing, but not movement self-consciousness, compared to Baseline (Zaback et al. 2015). Wong et al. (2009) showed that older adults who reported higher overall movement specific reinvestment were more accurate when responding to questions related to internal awareness. However, the authors did not distinguish between dimensions of MSRS. Therefore, we hypothesised that, when calculating the magnitude of relative change in each dimension of movement specific reinvestment (recorded as a state measure) between Baseline and Threat conditions, positive changes in both dimensions would be associated with improvements in internal monitoring and decrements in external monitoring. Finally, based on the assumption that older adult fallers increase reinvestment in an attempt to compensate for perceived deficits in balance ability (Wong et al. 2008, 2009), we predicted that the magnitude of the change in state anxiety and overall movement specific reinvestment (between Baseline and Threat conditions) would be negatively associated with a trait measure of balance confidence (i.e. older adults with lower balance confidence would be more vulnerable to heightened fall-related anxiety and reinvestment when facing increased postural threat).

## Methods

Twenty-four older adults completed 30 trials in a single session. All were able to walk unaided. Testing sessions took place in sheltered accommodation schemes in London, UK. Institutional ethical approval was obtained, all participants gave written and informed consent, and the protocol was carried out in accordance with the principles laid down by the Declaration of Helsinki.

Prior to the walking trials, participants completed: (a) the Montreal Cognitive Assessment (MoCA) (Nasreddine et al. 2005) (score <26/30 indicates mild cognitive impairment); (b) the Berg Balance Scale (Berg et al. 1997) (score <45/54 indicates significant functional balance impairment); (c) the Corsi Block test (Berch et al. 1998) (a paper test of visuospatial working memory, completed whilst seated); (d) the Digit Span test (Baddeley 1986, 2007) (a test of verbal working memory, completed whilst seated); (e) the Activities Balance Confidence Questionnaire (ABC) (Powell and Myers 1995). After completing these assessments, participants started the walking trials.

Participants walked at a comfortable pace over a six by four grid of 16 black and eight white paving blocks. The white blocks formed a nonlinear path, in which participants were instructed to navigate from one end to the other without stepping on the black blocks (Fig. 1). The blocks were constructed individually from solid wood (stepping surface = 40 cm^2^, height = 30 cm) and were fitted with non-slip rubber footers. The protocol was designed to mimic the common task of walking on a pavement, avoiding areas perceived to be unsafe. At the start of each trial, participants stood behind a screen (preventing them from seeing the walkway). When instructed to ‘Go’ participants walked around the screen, up a ramp (1200 mm long), along the white blocks and down a second ramp. An experimenter walked alongside participants to prevent them falling should they become unsteady. No instructions were given to participants other than to walk at a comfortable pace along the white path and to avoid stepping on the black blocks.

Participants walked the route in two experimental conditions: Baseline and Threat. In both conditions, participants completed five consecutive walks on one of three different routes (the order of which was randomised). The route was changed every five trials (15 trials in total for each Baseline and Threat condition). Directly after the first walk in each new route, participants were asked to complete a VSR task (i.e. recall the sequence of the white path, see Fig. 1a). During one of the following four trials on each route (randomly allocated), participants were asked a question whilst walking (e.g. ‘what did you have to eat and drink for breakfast this morning?’) and the experimenter observed if they SWWT. Twelve participants SWWT on at least one trial and were assigned to a SWWT group. The remaining twelve participants did not stop walking when asked a question on any trial and were assigned to a non-SWWT group (see Table 1 for participant characteristics). The equivalent number of participants in each group was not planned.

**Table 1:** Participant information

Measure (range of possible scores)	Non-SWWT (*n* = 12)	SWWT (*n* = 12)
Age (years)	75.8 ± 9.3	79.4 ± 5.4
Number of fallers (/12)^a^	4	6
Functional balance		
Berg balance test (0–54)	50.4 ± 3.5	49.6 ± 2.6
Balance confidence		
ABC (0–100)	73.2 ± 15.9	68.7 ± 14.8
Visual acuity (Snellen)	>20/40	>20/40
Cognitive function		
MoCA (0–30)	26.6 ± 2.9	26 ± 2.5
Reinvestment		
MSRS (0–40)	12.5 ± 9.8	18.2 ± 7.6*
Trait anxiety		
Spielberger’s trait anxiety inventory (20–80)	35.3 ± 7	42.6 ± 7*
State anxiety		
Spielberger’s state anxiety inventory (20–80)	35.3 ± 6.7	41.3 ± 7.6*
Time to complete walk (s)	5.53 ± 1.25	6.19 ± 2.64
External awareness		
Visuospatial recall (VSR) (−8 to +10)	5.7 ± 2.2	2.9 ± 2.1*
Internal awareness		
Number of correct responses (CR) (0–6)	2.5 ± 1.5	2.2 ± 1.2
Visuospatial working memory		
Corsi Block test (0–7)	3.9 ± 0.75	3.9 ± 0.67
Verbal working memory		
Digit Span test (0–8)	6.08 ± 0.70	5.50 ± 0.90

^a^Number of fallers represents the number of participants in each group who have fallen at least once in the 12 months prior to testing

*p < 0.05

**Fig. 2:**
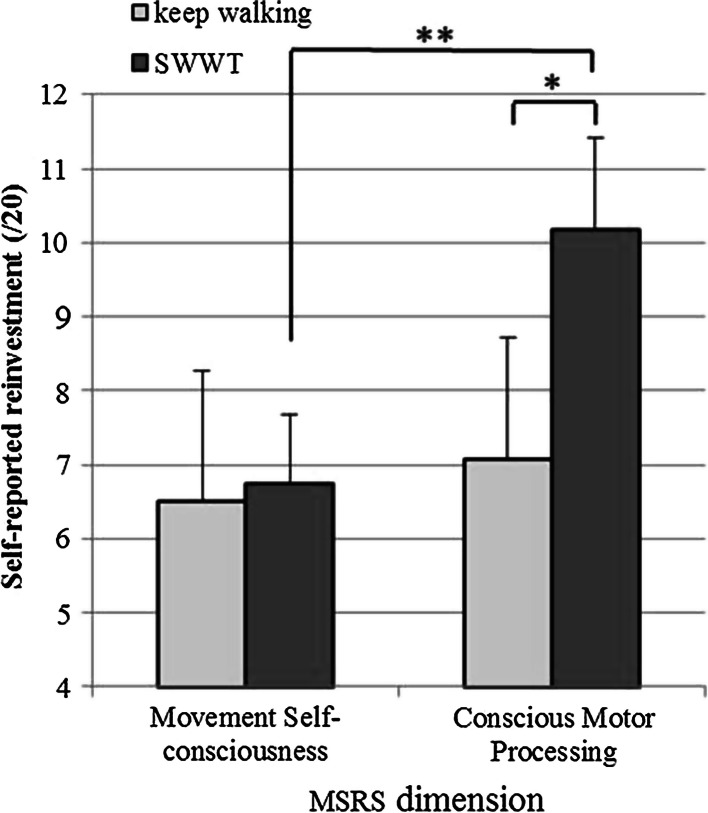
*Bar plot* showing scores from the two dimensions of the Movement Specific Reinvestment Scale: movement self-consciousness and conscious motor processing. *Error bars* represent standard error of the mean. *p < 0.05, **p < 0.005

Directly after one of the remaining trials (randomly allocated), participants were asked two questions relating to their ‘internal awareness’ (i.e. the position of their body at specific times during the walk) (e.g. ‘which of your feet did you first put on the first white block in the path?’ or ‘how many steps did you take on the first ramp?’). The total number of correct responses was recorded for both Baseline and Threat conditions (see Fig. 2). These questions were asked in only one of the five trials for each route in order to prevent participants from anticipating the questions after every trial and potentially monitoring their actions to a greater extent during each walk.

After completion of the trials for each route, participants returned behind the screen and completed shortened versions of both Spielberger’s State Anxiety Inventory (sh-SA) (Young et al. 2012) and the MSRS, which comprised of two questions related to movement self-consciousness (sh-MSRSms-c) and two related to conscious motor processing (sh-MSRScmp) (see Fig. 2 caption for descriptions). Participants completed each shortened questionnaire three times in each experimental condition (once for each route, from which the mean was calculated to represent state anxiety and reinvestment in each experimental condition). In addition to the shortened questionnaires, participants completed the full version of both Spielberger’s State Anxiety Inventory (SA) (Spielberger 1975) and the MSRS (Masters et al. 2005) directly after the Baseline walking trials. They were instructed to respond in terms of how they felt during the Baseline trials.

### Threat manipulation

In the Baseline condition, before the first trial on each route participants observed an experimenter walking on all of the blocks (black and white) to demonstrate that each was stable and safe to step on (although the instructions were to step on white blocks only). However, in the Threat condition, an experimenter walked on the blocks before the first trial on each route to demonstrate that, although the white blocks were stable, at least two black blocks (visually identical to all other blocks) would collapse if contacted by the participant’s foot (see Fig. 1b). The position of the collapsing blocks was described to participants as being randomised across trials. Viewing the collapsing blocks was intended to induce anxiety about the possibility of falling as a consequence of an inaccurate step. Prior to each trial, participants were asked to stand behind the screen whilst the experimenter positioned the collapsing blocks in a random location. In fact, the experimenter replaced the collapsible blocks with solid blocks, so that participants were (unwittingly) never at risk of stepping on a collapsible block.

In order to remove any doubt concerning the presence of collapsible blocks, we included one false trial in which an experimenter caused a black block to collapse by pulling a nylon wire (hidden from the participant’s view), which released magnets in the block mechanism causing it to collapse during the participant’s approach to the ramp (see Route 2, Fig. 1a). Once the block collapsed, an experimenter stopped the trial and explained that this was an equipment malfunction and the trial would be repeated after the black blocks were rearranged. Participants all completed trials in the Threat condition after Baseline trials. This procedure was undertaken to avoid participants being fearful of the blocks falling during Baseline trials.

### SWWT group comparisons for Baseline trials

Separate one-way ANOVAs were used to identify differences between SWWT and non-SWWT groups for the following variables: (a) MSRS; (b) Trait and State anxiety inventories; (c) MoCA; (d) VSR; (e) Berg Balance Scale; (f) time to complete the walking task. To identify whether SWWT differed between MSRS dimensions, a repeated-measures ANOVA was used with a 2 × 2 design (SWWT group × MSRS dimension). In order to establish if any relationship between reinvestment and SWWT was attributable to between-subject differences in general cognitive function, a one-way ANCOVA was conducted on both MSRSms-c and MSRScmp scores using the MoCA as a covariate. Two independent samples Mann–Whitney *U* tests were performed on internal awareness scores (to determine between-group differences in propensity to focus attention internally during the task) (Wong et al. 2008, 2009) and on Corsi Block and Digit Span test scores to determine between-group differences in the relevant aspects of working memory. These nonparametric tests were necessary because of the low number of possible scores that could be achieved on the internal awareness (0–6), Corsi Block (0–7) and Digit Span (0–8) tests, effectively rendering each of these dependent variables an ordinal scale.

No statistical analysis was carried out on the frequency or duration of SWWT. With regard to the frequency of SWWT, the majority of participants either SWWT in none or all of the trials. Two participants SWWT in the first path sequence (Baseline condition) and then did not SWWT in the remaining five path sequences. These participants were assigned to the non-SWWT group.

### Baseline versus threat comparisons

**Fig. 3:**
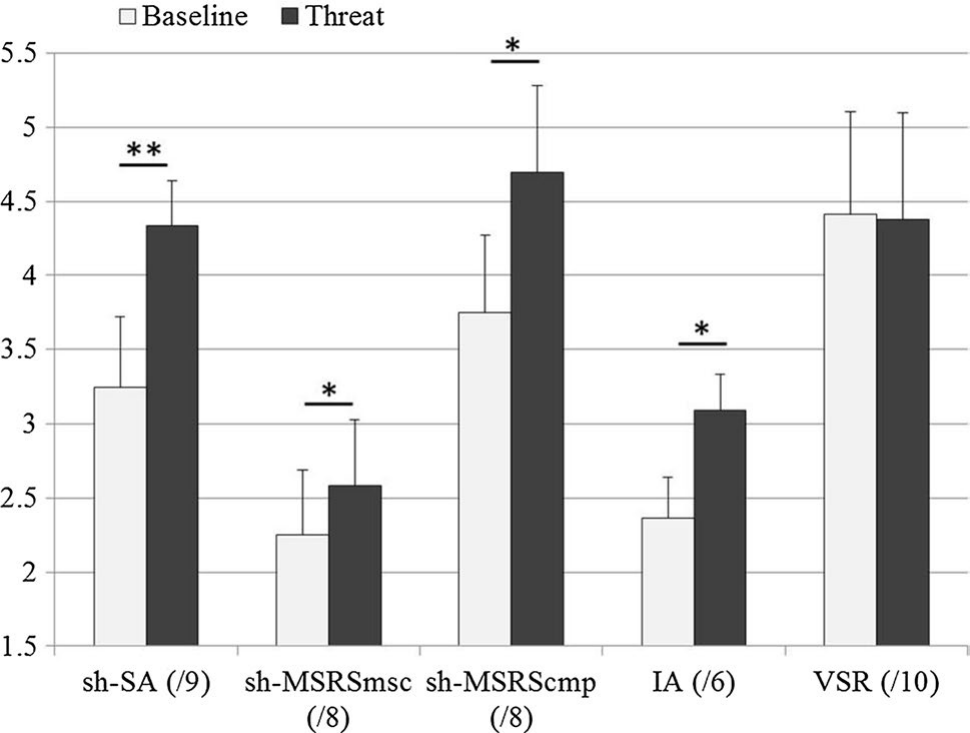
(SA) Shortened version of Spielberger’s State anxiety questionnaire (Spielberger 1975). Three items: (1) I feel calm when completing the task (negatively scored as per original questionnaire); (2) I feel tense when completing the task; (3) I am worried that I may lose my balance. Possible responses were: 0 = Not at all; 1 = Somewhat; 2 = Moderately; 3 = Very much (maximum range of possible scores = 0–9). (MSRS) Shortened version of the Movement Specific Reinvestment Scale comprised two items from each dimension of *movement self*-*consciousness* (sh-MSRSmsc): (1) I am self-conscious about the way I look when I am moving; (2) I sometimes have the feeling that I am watching myself move, and *conscious motor processing* (sh-MSRScmp): (1) I am always trying to think about my movements when I carry them out; (2) I am aware of the way my body moves when I carry out a movement. These four items were selected from the total of 10 items in the original MSRS scale as they were deemed by the authors to be most strongly suited to measure state reinvestment during gait. Possible responses were: 0 = Extremely uncharacteristic; 1 = Uncharacteristic; 2 = Neutral; 3 = Characteristic; 4 = Extremely characteristic (maximum range of possible scores = 0–8 for each factor). For measures of sh-SA, sh-MSRSmsc and sh-MSRScmp, scores represent the mean of three administrations of each questionnaire after the final trial within each path sequence, in both Baseline and Threat conditions. *Error bars* represent standard error of the mean. *p < 0.05, **p < 0.005

Related-samples Wilcoxon signed-rank tests were used to determine significant changes between Baseline and Threat conditions for measures of sh-SA, sh-MSRSms-c, sh-MSRScmp and internal awareness. These nonparametric tests were necessary because of the low number of possible scores that could be achieved for sh-SA (0–9), sh-MSRSms-c (0–8) and sh-MSRScmp (0–8) (see Fig. 3 caption). In contrast, the range of possible scores in the VSR test was −8 to +10 in increments of 0.5 (permitting 35 possible outcome scores) (see Fig. 1 caption), so a paired-samples *t* test was used to compare VSR scores between Baseline and Threat conditions.

In order to determine if balance confidence was associated with the magnitude of change in sh-SA or state reinvestment, state scores for both sh-SA and reinvestment (combined score for sh-MSRSms-c and sh-MSRScmp questions) at Baseline were subtracted from scores recorded in the Threat condition and correlated with self-reported ABC scores using Spearman’s rank correlation coefficient. Significance levels were set a priori at *p* < 0.05.

## Results

### SWWT group comparisons for Baseline trials

The SWWT group reported significantly higher MSRS scores compared to non-SWWT [*F*(1,23) = 5.928, *p* = 0.023, *η*_p_^2^ = .212]. The SWWT group also reported significantly higher trait, *F*(1,23) = 7.702, *p* = 0.017, *η*_p_^2^ = .242, and state anxiety *F*(1,23) = 7.030, *p* = 0.016, *η*_p_^2^ = .206 (see Table 1).

Breaking down MSRS scores into two dimensions (movement self-consciousness and conscious motor processing) revealed a two-way interaction between SWWT and MSRS dimension [*F*(1,22) = 5.230, *p* = 0.032, *η*_p_^2^ = .192]. Post hoc analysis showed no differences between groups in the dimension of MSRSms-c. However, there was a significant between-group difference in MSRScmp [*F*(1,23) = 6.125, *p* = 0.022, *η*_p_^2^ = .218]. Furthermore, comparisons between MSRS dimensions within each group showed that MSRScmp was significantly higher compared to MSRSms-c, but only in the SWWT group [*t*(11), −4.326, *p* = 0.001] (see Fig. 2).

ANCOVA showed that the significant group differences in overall MSRS and MSRScmp were both preserved when MoCA scores were included as a covariate [*F*(1,23) = 7.488, *p* = 0.012, *η*_p_^2^ = .066 and *F*(1,23) = 5.612, *p* = 0.031, *η*_p_^2^ = .260, respectively]. There was no between-group difference in MoCA scores (see Table 1), indicating that age-related decline in cognitive function did not significantly influence SWWT, nor did it exacerbate our proposed influence of reinvestment on SWWT. There were no significant differences in ABC scores or the time to complete the walking task (see Table 1).

Older adults who SWWT scored worse on the VSR task [*F*(1,23) = 9.459, *p* = 0.005, *η*_p_^2^ = .342], suggesting that attention was allocated away from external task-relevant information. There was no significant between-group difference in scores for the Corsi Block or Digit Span tests (see Table 1), indicating that group differences in VSR were unlikely to have resulted from trait differences in working memory capacity. There was no significant difference between groups for internal awareness. However, Spearman’s rank correlation coefficient showed a significant correlation between MSRSms-c and internal awareness [*r*(24) = .435, *p* < 0.05], but not VSM. There were no significant correlations between MSRScmp, internal awareness and VSM.

### Baseline versus Threat comparisons

During trials containing the Threat condition, reports of sh-SA (*Z* = −2.603, *p* < 0.05), sh-MSRSms-c (*Z* = −2.138, *p* < 0.05), sh-MSRScmp (*Z* = −3.002, *p* < 0.005) and internal awareness (*Z* = −2.416, *p* < 0.01) were significantly higher than Baseline (see Fig. 3). There was no significant effect of Threat on VSM or time taken to complete the walk.

Spearman’s rank correlation coefficient showed significant negative correlations between ABC scores and the degree to which sh-SA and overall state reinvestment scores increased between Baseline and Threat conditions [*r*(24) = −.551, *p* < 0.01 and *r*(24) = −.431, *p* < 0.05, respectively]. These moderate correlations indicate that levels of sh-SA and reinvestment in older adults with lower balance confidence were more susceptible to increase in the Threat condition compared to those with higher balance confidence.

## Discussion

We examined whether older adults who SWWT self-report a relatively higher propensity to consciously control walking actions compared to: (1) older adults who do not SWWT; and (2) a propensity for movement self-consciousness when walking.

Our findings show that older adults who SWWT self-report higher reinvestment compared to non-SWWT, but only in the dimension of conscious motor processing, not movement self-consciousness (Fig. 2). Researchers have previously rationalised SWWT according to so-called age-related shifts in the allocation of generic attentional resources (Ayers et al. 2014; Boisgontier et al. 2013; Verghese et al. 2007, 2008), but our findings suggest that the phenomenon is specific to aspects of working memory engaged in responding to a question when walking. We propose that a propensity to consciously control walking actions puts older adults in a situation where they must prioritise between walking and talking tasks due to competition for common resources in verbal aspects of working memory. Conversely, those who do not consciously control their movements to the same extent can perform both tasks concurrently, by virtue of the relative automaticity of locomotion.

We predicted that the ability to consciously control gait whilst concurrently responding to a question would depend on levels of general cognitive function. Ayers et al. (2014) speculated that gait dysfunction during walking when talking tasks is potentially an early indicator of cognitive impairments (Verghese et al. 2008). However, not only did our results show an absence of any significant between-group difference in MoCA scores, but also between-group differences in MSRScmp (Fig. 2) were independent of MoCA scores, indicating that the relationship between SWWT and conscious motor processing is not strongly mediated by age-related cognitive deficits. The processes underpinning SWWT appear to be indicative of ‘structural interference’ between the two tasks, suggesting that the propensity to conscious control movements may have a polarising influence on SWWT behaviour, regardless of general cognitive function.

### Internal versus external awareness

We report a significant correlation between MSRSms-c (but not MSRScmp) and internal awareness scores, leading us to the conclusion that self-reported increases in movement self-consciousness were associated with increased internal awareness. Previously, researchers have shown that older adult fallers (who report high overall MSRS scores) make more accurate responses in similar measures of internal awareness (Wong et al. 2008, 2009). Our findings extend this perspective, demonstrating differential associations between each dimension of the MSRS and specific aspects of older adults’ allocation of attention during adaptive gait (i.e. MSRSms-c reflects internal awareness and MSRScmp relates to verbally processing explicit rules for movement execution and related SWWT behaviour).

As predicted, the results showed that VSR scores were significantly worse (approximately halved) in the SWWT group compared to non-SWWT; differences that occurred despite an absence of any between-group contrasts in the trait capacity to retain visuospatial and verbal information in working memory. We propose that these pronounced reductions in VSR scores reflect allocation of attention away from task-relevant external factors. Given that MSRSms-c and internal awareness scores were highly comparable between the SWWT and non-SWWT group, it seems likely that conscious motor processing was prioritised in older adults who SWWT at the cost of external awareness. These results are somewhat contradictory of those described by Wong et al. (2008, 2009) who found no significant between-group differences in the accuracy of answers to questions deemed to reflect external awareness. The between-group differences shown in the current study are likely to pertain to our alternative distinction used to categorise groups (i.e. SWWT rather than fall-status). However, it is likely that our VSR method better reflects a walker’s awareness of factors that are exclusively external and task-relevant. Collectively, these findings are indicative of behaviours that have worrying consequences for the likelihood of falls in older adults who consciously control movements. For example, the reduced VSR scores described here may be associated with reductions in the efficiency of visual previewing of an intended path; behaviour observed in anxious older adults that is associated with increased frequency of gross stepping errors (Young et al. 2012; Young and Hollands 2012; Young and Williams 2015).

### Baseline versus Threat comparisons

The second objective of the current study was to establish if anxiety is causally related to reinvestment in the context of adaptive gait in older adults. We predicted that our anxiety manipulation would lead to significant increases in state anxiety, both dimensions of reinvestment and internal awareness. In the Threat condition, significant increases were evident in sh-SA, sh-MSRScmp, sh-MSRSms-c and internal awareness (Fig. 3). As previously speculated by several researchers (Wong et al. 2008, 2009; Masters and Maxwell 2008; Young and Williams 2015), the current results support claims of a causal relationship between anxiety and reinvestment in older adults when walking. These findings are reminiscent of those described by Nibbeling et al. (2012), who asked young adults to run on a treadmill at different heights above ground level. When running at height, participants allocated attention to processing thoughts relevant to postural threat at a cost to efficient running (Nibbeling et al. 2012). The current results extend previous observations in young adults performing stationary posture (Huffman et al. 2009) and walking (Gage et al. 2003) tasks to older adults performing an adaptive gait task that is broadly representative of tasks encountered in daily life.

Heightened fall-related anxiety observed in the Threat condition was associated with increased self-reported movement self-consciousness and conscious motor processing (Fig. 3), but we are cautious about assuming a uni-directional relationship in which both dimensions of reinvestment are directly regulated by levels of performance anxiety. Several researchers have shown that adopting an internal focus of attention can contribute to, and maintain, emotional disorders and attentional bias for threat, which in turn can propagate anxiety (Coombes et al. 2009; Pfleiderer et al. 2014). This latter perspective is supported by neurological evidence showing that internal awareness is associated with activity in fear-relevant areas of the brain (e.g. amygdala, thalamus, parahippocampus; Domschke et al. 2010; Paulus and Stein 2010). The perceived threat of accidentally stepping on an unstable block may have caused pragmatic attempts to ensure task success via movement specific reinvestment. The initiation of this process may have been independent from anxiety. However, as suggested by the Psychophysiological Model of Panic (Ehlers et al. 1995), drawing attention to internal factors can heighten perceptions of physiological change (such as increased heart rate or muscle tension), which can be wrongly interpreted as fear-related responses to a perceived danger (i.e. the threat of a falling block). This interpretation may explain the significant negative correlations between balance confidence and the magnitude of increase in both state anxiety and reinvestment scores in Threat compared to Baseline trials. Further work is required to clarify these mechanisms in the context of fall-related anxiety in older adults, and to evaluate the influence of specific trait difference between individuals, such as fear of falling and balance confidence.

The magnitude of increases in anxiety and related reinvestment observed in the Threat condition were seemingly not sufficient to compromise external awareness or significantly increase the time taken to complete the task. The walking task was designed to be highly demanding and broadly representative of situations encountered in the daily lives of community-dwelling older adults. Without measuring specific gait parameters or objective measures of balance control, we cannot conclude that balance control was altered in the Threat condition compared to Baseline. However, if the demands of the walking task were progressively increased, one would expect that increased anxiety would eventually lead to gait disturbances. This assumption is based on Attentional Control Theory (Eysenck et al. 2007), which predicts that anxiety causes an attentional bias towards threat-related and/or task-irrelevant stimuli, resulting in reduced attentional processing efficiency (Eysenck and Calvo 1992; Eysenck et al. 2007). If the execution of a highly automated action, such as walking, requires the use of conscious monitoring and/or guidance, any associated cognitive demands might be considered a task-irrelevant distraction (Lam et al. 2009) as they consume cognitive resources that could otherwise be used to avoid inefficiencies in the primary task, such as SWWT. According to Attentional Control Theory (Eysenck et al. 2007), and its predecessor Processing Efficiency Theory (Eysenck and Calvo 1992), providing the demands of the primary task are low, anxiety-related inefficiencies in working memory can be compensated for by increasing mental effort. However, if task difficulty is progressively increased, there will be a point at which increased effort can no longer compensate for anxiety-related reductions in processing efficiency, and performance in the primary task (e.g. walking) will decline. This hypothesis is supported by a recent review by Boisgontier et al. (2013) who found that there is little evidence to support claims for age-related increases in the attentional demands of simple forward walking tasks (also see Coombes et al. 2009). However, such age-related demands clearly emerge in more demanding dynamic tasks involving unstable support surfaces (Boisgontier et al. 2013). Our findings suggest that it may be misleading to consider age-related increases in the attentional demands of controlling gait to be a general rule. Future work must endeavour to differentially associate specific aspects of cognition with various gait disturbances observed in older adults, particularly those that predict falls, such as SWWT.

Donoghue et al. (2013) suggested that fear of falling can influence older adults by causing (1) increased vigilance, which leads to cautious behaviour and (2) activity avoidance. Our results potentially identify a possible third consequence of fear of falling in which anxiety-related increases in reinvestment (particularly conscious motor processing) place demands on valuable cognitive resources, causing reduced external awareness and gait disturbances (e.g. SWWT) that are in turn predictive of fall-risk (Lundin-Olsson et al. 1997; Beauchet et al. 2009). Whilst acknowledging the limitation of a small sample size, our results showed a significant negative correlation between the magnitude of increase observed in sh-SA from Baseline to Threat conditions and trait ABC scores. Consequently, older adults with low balance confidence may be particularly vulnerable to anxiety-related inefficiencies in attentional processing, potentially placing them at greater risk of falling when faced with demanding gait tasks (Young and Williams 2015).

The sample size included in the current study is relatively small and represents a significant limitation. Whereas the sample was sufficient to demonstrate differences between SWWT and non-SWWT groups in addition to certain threat-related changes, certain comparisons are susceptible to type II error. For example, based on the power of the SWWT test in predicting falls (Lundin-Olsson et al. 1997; Beauchet et al. 2009), one would expect to find significant between-SWWT-group differences in ABC, the time taken to complete the walking task, and any variable commonly associated with fall-risk (Ayers et al. 2014; Verghese et al. 2008). A clear association existed between self-reported conscious motor processing and SWWT, which highlights the importance of further examining these and other psychological processes associated with reinvestment (e.g. propensity for risk-taking, Zaback et al. 2015; Butler et al. 2015). In future, researchers should also examine whether associations exist between these psychological factors and specific gait characteristics/relevant behaviours, such as visual search.

## Conclusions

We highlighted an association between SWWT and the self-reported propensity of older adults to consciously control their walking, an association that appears to be independent from levels of general cognitive function. Older adults who SWWT scored markedly worse on the VSR test, suggesting that these older adults were allocating attention away from task-relevant external factors towards internal conscious movement control. Self-reported movement self-consciousness was no different between SWWT groups, yet this measure was associated with internal awareness. The results extend the current literature by implicating specific attentional processes that are likely to contribute to significant gait disturbances, especially during adaptive and demanding gait tasks.

To our knowledge, this is the first occasion on which anxiety has been successfully manipulated during a complex adaptive gait task. Our anxiety manipulation showed that both movement self-consciousness and conscious motor processing (and associated internal awareness scores) are causally associated with fall-related anxiety, and that these relationships exist in the context of older adults performing a complex adaptive gait task that is broadly representative of locomotion tasks encountered in daily life. Further work is necessary to establish specific ways in which both dimensions of movement specific reinvestment might influence fall-risk, be it detrimental or beneficial.
